# Mobile genomic element diversity in world collection of safflower (*Carthamus tinctorius* L.) panel using iPBS-retrotransposon markers

**DOI:** 10.1371/journal.pone.0211985

**Published:** 2019-02-26

**Authors:** Fawad Ali, Abdurrahim Yılmaz, Muhammad Azhar Nadeem, Ephrem Habyarimana, Ilhan Subaşı, Muhammad Amjad Nawaz, Hassan Javed Chaudhary, Muhammad Qasim Shahid, Sezai Ercişli, Muhammad Abu Bakar Zia, Gyuhwa Chung, Faheem Shehzad Baloch

**Affiliations:** 1 Department of Field Crops, Faculty of Agricultural and Natural Science, Bolu Abant Izzet Baysal University, Bolu, Turkey; 2 Department of Plant Sciences, Quaid-I- Azam University, Islamabad, Pakistan; 3 Consiglio per la ricerca in agricoltura e l'analisi dell'economia agraria–Centro di ricerca cerealicoltura e colture industriali, Bologna, Italy; 4 Central Research Institute for Field Crops, Ankara, Turkey; 5 Education Scientific Center of Nanotechnology, Far Eastern Federal University, Vladivostok, Russian Federation; 6 State Key Laboratory for Conservation and Utilization of Subtropical Agro-bio resources, South China Agricultural University, Guangzhou, China; 7 Department of Horticulture, Faculty of Agriculture, Ataturk University, Erzurum, Turkey; 8 Department of Plant breeding and genetics, Muhammad Nawaz Sharif University of Agriculture, Multan, Pakistan; 9 Department of Biotechnology, Chonnam National University, Chonnam, Republic of Korea; Nigde Omer Halisdemir University, TURKEY

## Abstract

Safflower (*Carthamus tinctorius* L.) is a multipurpose crop of dry land yielding very high quality of edible oil. Present study was aimed to investigate the genetic diversity and population structure of 131 safflower accessions originating from 28 different countries using 13 iPBS-retrotransposon markers. A total of 295 iPBS bands were observed among which 275 (93.22%) were found polymorphic. Mean Polymorphism information content (0.48) and diversity parameters including mean effective number of alleles (1.33), mean Shannon’s information index (0.33), overall gene diversity (0.19), Fstatistic (0.21), and inbreeding coefficient (1.00) reflected the presence of sufficient amount of genetic diversity in the studied plant materials. Analysis of molecular variance (AMOVA) showed that more than 40% of genetic variation was derived from populations. Model-based structure, principal coordinate analysis (PCoA) and unweighted pair-group method with arithmetic means (UPGMA) algorithms clustered the 131 safflower accessions into four main populations A, B, C, D and an unclassified population, with no meaningful geographical origin. Most diverse accessions originated from Asian countries including Afghanistan, Pakistan, China, Turkey, and India. Four accessions, Turkey3, Afghanistan4, Afghanistan2, and Pakistan24 were found most genetically distant and might be recommended as a candidate parents for breeding purposes. The findings of this study are most probably supported by the seven similarity centers hypothesis of safflower. This is a first study to explore the genetic diversity and population structure in safflower accessions using the iPBS-retrotransposon markers. The information provided in this work will therefore be helpful for scientists interested in safflower breeding.

## 1. Introduction

Safflower (*Carthamus tinctorius* L.) belongs to the *Compositae* family, it is self-pollinated and has a haploid genome size of about 1.4 GB and 2n = 24 chromosomes [1]. This crop is cultivated over wide geographical zones throughout the world for several uses including production of dyes, extraction of edible oil, and various medicinal utilizations [2]. Safflower has been in use since ancient times and the archeological remains of *Carthamus* spp. were found at sites in Syria about 7500 BC ago [3]. From these sites, cultivation of safflower spread to other regions like, Egypt, the Aegean, and southeastern Europe.

Safflower accessions specific to some regions show similarity on the basis of their morphological traits and such a region can be considered as a safflower similarity center. However, there is still a debate on the actual number of similarity centers in the world as ascertained by molecular markers [4]. Knowles [5] proposed seven similarity centers (1: Far East, 2: India-Pakistan, 3: Middle East, 4: Egypt, 5: Sudan, 6: Ethiopia, and 7: Europe) for safflower while, Ashri [6] identified ten similarity centers (1: Near East, 2: Iran/Afghanistan, 3: Turkey, 4: Egypt, 5: Ethiopia, 6: Sudan, 7: Far East, 8: India/Pakistan, 9: Europe, and 10: Kenya). Similarly, Chapman et al. [4] proposed five safflower similarity centers for safflower (1: Near East, 2: Iran & Afghanistan, Turkey, 3: Egypt, Ethiopia, (Sudan), 4: Far East, India/Pakistan, (Sudan), 5: Europe).

It is estimated that safflower is cultivated in nearly 20 countries with a total cultivated area of 1,140,002 hectares and the production of 948,516 tons [7]. Safflower major producer countries include, Russian Federation (286,351 tons), Kazakhstan (167,243 tons), Mexico (121,767 tons), USA (99,830 tons), Turkey (58,000 tons), and India (53,000 tons) which account for about 71% of the total world production [7]. In spite of containing good amount of polyunsaturated fatty acids and being resistant to dry conditions, still safflower did not gain the status of major oilseed crop. The primary factors which prevented its cultivation on large scale are low seed yield, low oil content, biotic stresses susceptibility, and spininess [8]. Therefore, the enhanced acceptability and utilization of safflower as an oilseed crop will require genetic improvement for the traits of interest. To this end, genetic diversity can be an effective approach by providing a good source of variations upon which breeding programs can build [9]. However, it is unfortunate that current safflower germplasm and breeding lines displayed low levels of genetic diversity, and were therefore of reduced usefulness in breeding programs. An extensive genetic and phenotypic diversity characterization among global safflower germplasm can help broaden the genetic base and diversity in the safflower crop, and identify elite accessions [10–11]. Safflower genetic diversity was investigated using different molecular markers; Random Amplified Polymorphic DNA (RAPD), Inter Simple Sequence Repeat (ISSR), Amplified Fragment Length Polymorphism (AFLP), Simple Sequence Repeats (SSRs), and Single Nucleotide Polymorphism (SNPs) [4–11–12–13–14–15–16–17–18–19–20], but so far, iPBS-retrotransposon markers have not been used to investigate the genetic diversity in safflower.

Retrotransposons are known as an important component of the plant genome in terms of structural evolution and have great potential of changing its position and copy number across plant genome [21]. Retrotransposons are genetic elements ranging from 50 to 90% in various plant genomes depending upon the plant species [22]. Long terminal repeat (LTR) and non- long terminal repeat (non-LTR) are the two classes of retrotransposons, and plant genome reveal higher proportions of LTR retrotransposons as compare to non-LTR [23]. Limitations in the retrotransposon marker systems resulted in the development of a new marker system named Inter-primer binding site (iPBS) retrotransposons having universal applicability [23–24]. iPBS is a PCR-based, universal marker system and depends upon the presence of tRNA as a reverse transcriptase primer binding site [25]. Minimum cost and high efficiency of iPBS-retrotransposons make them good marker system [23]. Various crops like, pea, chickpea, *Lens*, Turkish okra, Tobacco, and common bean have been studied efficiently using iPBS-retrotransposon markers system [26–27–28–29–30].

Several crop species have been improved utilizing molecular markers in various crop breeding programs [31]. However, for safflower, its genetics and genomics were less studied, which can explain the lack of reliable marker systems for use in the process of developing superior safflower cultivars [32–33]. This study was conducted to evaluate the genetic diversity and population structure of safflower accessions using iPBS-retrotransposons as a start for further scientific investigations and practical breeding use cases.

## 2. Materials and Methods

### 2.1. Plant materials and DNA isolation

Experimental materials comprising 131 safflower accessions collected from 28 different countries were evaluated in this study. Among these accessions, 94, 17, and 20 originated from the United States Department of Agriculture (USDA), Plant Genetic Resources Institute Pakistan, and from the Turkish Central Research Institute for Field Crops (Table 1). A total of 94 accessions from USDA and 17 from Pakistan used in this study were landraces. The 20 Turkish accessions were single plant selection among international germplasm from USDA and are candidate cultivars. Seeds of each accession were sown at the research and experimental area of Bolu Abant Izzet Baysal University. Fresh, young healthy leaves were harvested at proper time for the isolation of DNA, brought to laboratory and frozen at -80°C for later use. DNA extraction was performed using the bulk leaves of each accession, and followed CTAB protocol [34] with slight modifications [35]. DNA concentration of each accession was measured using agarose gel (0.8%) and was also confirmed with the help of NanoDrop (DeNovix DS-11 FX, USA). Final DNA concentration for the 131 accession samples to be used in polymerase chain reactions (PCR) was adjusted to 5 ng/μL; the samples were stored at -25 ^o^C till the start of PCR amplifications.

**Table 1:** Passport data of 131 world safflower panel.

Accession Number	Genotype Name	Accession No	Donor Organization	Location	Province/District	Country Origin	Plant ID	Continent
G1	Isreal-1	30548	USDA	-	-	Isreal	P1-198990	Asia
G2	Romania-1	30549	USDA	-	-	Romania	P1-209287	Europe
G3	Morocco-1	30552	USDA	-	-	Morocco	P1-239042	Africa
G4	Egypt-1	30563	USDA	-	-	Egypt	P1-250082	Africa
G5	Pakistan-1	30564	USDA	-	-	Pakistan	P1-250194	Asia
G6	Pakistan-2	30565	USDA	-	-	Pakistan	P1-250201	Asia
G7	Pakistan-3	30567	USDA	-	-	Pakistan	P1-250345	Asia
G8	Pakistan-4	30568	USDA	-	-	Pakistan	P1-250346	Asia
G9	Pakistan-5	30569	USDA	-	-	Pakistan	P1-250351	Asia
G10	Pakistan-6	30570	USDA	-	-	Pakistan	P1-250353	Asia
G11	Pakistan-7	30573	USDA	-	-	Pakistan	P1-250481	Asia
G12	Egypt-2	30574	USDA	-	-	Egypt	P1-250528	Africa
G13	Egypt-3	30577	USDA	-	-	Egypt	P1-250532	Africa
G14	Egypt-4	30578	USDA	-	-	Egypt	P1-250540	Africa
G15	India-1	30579	USDA	-	-	India	P1-250601	Asia
G16	Egypt-4	30580	USDA	-	-	Egypt	P1-250605	Africa
G17	Egypt-6	30581	USDA	-	-	Egypt	P1-250608	Africa
G18	Iran-1	30588	USDA	-	-	Iran	P1-250720	Asia
G19	Jordan-1	30589	USDA	-	-	Jordan	P1-251284	Asia
G20	Jordan-2	30590	USDA	-	-	Jordan	P1-251285	Asia
G21	Isreal-2	30594	USDA	-	-	Isreal	P1-253386	Asia
G22	Spain-1	30595	USDA	-	-	Spain	P1-253388	Europe
G23	Spain-2	30596	USDA	-	-	Spain	P1-253391	Europe
G24	Spain-3	30597	USDA	-	-	Spain	P1-253394	Europe
G25	Spain-4	30598	USDA	-	-	Spain	P1-253395	Europe
G26	Portugal-1	30604	USDA	-	-	Portugal	P1-253553	Europe
G27	Portugal-2	30605	USDA	-	-	Portugal	P1-253556	Europe
G28	Morocco-2	30606	USDA	-	-	Morocco	P1-253560	Africa
G29	Portugal-3	30608	USDA	-	-	Portugal	P1-253564	Europe
G30	Portugal-4	30610	USDA	-	-	Portugal	P1-253569	Europe
G31	Portugal-5	30611	USDA	-	-	Portugal	P1-253571	Europe
G32	Iraq-1	30612	USDA	-	-	Iraq	P1-253761	Asia
G33	Iraq-2	30613	USDA	-	-	Iraq	P1-253762	Asia
G34	Afghanistan-1	30614	USDA	-	-	Afghanistan	P1-253764	Asia
G35	Isreal-3	3015	USDA	-	-	Isreal	P1-253892	Asia
G36	Syria-1	30616	USDA	-	-	Syria	P1-253898	Asia
G37	Syria-2	30617	USDA	-	-	Syria	P1-253900	Asia
G38	Portugal-6	30620	USDA	-	-	Portugal	P1-258412	Europe
G39	Uzbekistan-1	30623	USDA	-	-	Uzbekistan	P1-262435	Asia
G40	China-1	30624	USDA	-	-	China	P1-262452	Asia
G41	China-2	30625	USDA	-	-	China	P1-262453	Asia
G42	Iran-2	30631	USDA	-	-	Iran	P1-304444	Asia
G43	Iran-3	30633	USDA	-	-	Iran	P1-304448	Asia
G44	Turkey-1	30646	USDA	-	-	Turkey	P1-304498	Asia
G45	Turkey-2	30648	USDA	-	-	Turkey	P1-304502	Asia
G46	Turkey-3	30650	USDA	-	-	Turkey	P1-304504	Asia
G47	Turkey-4	30651	USDA	-	-	Turkey	P1-304505	Asia
G48	Afghanistan-2	30653	USDA	-	-	Afghanistan	P1-304592	Asia
G49	India-2	30662	USDA	-	-	India	P1-305195	Asia
G50	Russia-1	30663	USDA	-	-	Russia	P1-305535	Asia
G51	India-3	30673	USDA	-	-	India	P1-306926	Asia
G52	India-4	30674	USDA	-	-	India	P1-306941	Asia
G53	India-5	30677	USDA	-	-	India	P1-306976	Asia
G54	Kazakhstan-1	30681	USDA	-	-	Kazakhstan	P1-314650	Asia
G55	Turkey-5	30688	USDA	-	-	Turkey	P1-340086	Asia
G56	Argentina-1	30695	USDA	-	-	Argentina	P1-367833	America
G57	Uzbekistan-2	30696	USDA	-	-	Uzbekistan	P1-369846	Asia
G58	Uzbekistan-3	30697	USDA	-	-	Uzbekistan	P1-369853	Asia
G59	Syria-3	30700	USDA	-	-	Syria	P1-386174	Asia
G60	Thailand-1	30701	USDA	-	-	Thailand	P1-387821	Asia
G61	Iran-4	30713	USDA	-	-	Iran	P1-405958	Asia
G62	Iran-5	30718	USDA	-	-	Iran	P1-405967	Asia
G63	Bangladesh-1	31509	USDA	-	-	Bangladesh	PI-401472	Asia
G64	Bangladesh-2	31510	USDA	-	-	Bangladesh	PI-401478	Asia
G65	Bangladesh-3	31511	USDA	-	-	Bangladesh	PI-401480	Asia
G66	India-6	33538	USDA	-	-	India	PI 199878	Asia
G67	Afghanistan-3	33541	USDA	-	-	Afghanistan	PI 220647	Asia
G68	Australia-1	33542	USDA	-	-	Australia	PI 235660	Oceania
G69	Turkey-6	33543	USDA	-	-	Turkey	PI 237538	Asia
G70	Pakistan-8	33547	USDA	-	-	Pakistan	PI 250474	Asia
G71	Pakistan-9	33548	USDA	-	-	Pakistan	PI 250478	Asia
G72	Iran-6	33556	USDA	-	-	Iran	PI 250840	Asia
G73	Jordan-3	33559	USDA	-	-	Jordan	PI 251265	Asia
G74	Jordan-4	33560	USDA	-	-	Jordan	PI 251267	Asia
G75	Jordan-5	33561	USDA	-	-	Jordan	PI 251268	Asia
G76	Israel-4	33564	USDA	-	-	Israel	PI 251290	Asia
G77	Turkey-7	33565	USDA	-	-	Turkey	PI 251978	Asia
G78	Turkey-8	33567	USDA	-	-	Turkey	PI 251984	Asia
G79	Austria-1	33568	USDA	-	-	Austria	PI 253519	Europe
G80	Hungary-1	33575	USDA	-	-	Hungary	PI 288983	Europe
G81	Libya-1	33608	USDA	-	-	Libya	PI 393499	Africa
G82	Bangladesh-4	33609	USDA	-	-	Bangladesh	PI 401470	Asia
G83	Iran-7	33621	USDA	-	-	Iran	PI 406010	Asia
G84	Turkey-9	33627	USDA	-	-	Turkey	PI 406701	Asia
G85	Turkey-10	33628	USDA	-	-	Turkey	PI 406702	Asia
G86	Pakistan-10	33635	USDA	-	-	Pakistan	PI 426521	Asia
G87	China-3	33638	USDA	-	-	China	PI 543979	Asia
G88	China-4	33639	USDA	-	-	China	PI 543982	Asia
G89	China-5	33642	USDA	-	-	China	PI 544001	Asia
G90	China-6	33651	USDA	-	-	China	PI 568809	Asia
G91	China-7	33661	USDA	-	-	China	PI 568874	Asia
G92	France-1	33662	USDA	-	-	France	PI 576985	Europe
G93	Austria-2	33670	USDA	-	-	Austria	BVAL-901352	Europe
G94	Pakistan-11	Check	PGRI-Pakistan	-	-	Pakistan	Thori-78	Asia
G95	Pakistan-12	16266	PGRI-Pakistan	Jacobabad	Sindh	Pakistan	-	Asia
G96	Pakistan-13	16267	PGRI-Pakistan	Shikarpur	Sindh	Pakistan	-	Asia
G97	Pakistan-14	16268	PGRI-Pakistan	Shikarpur	Sindh	Pakistan	-	Asia
G98	Pakistan-15	16269	PGRI-Pakistan	Larkana	Sindh	Pakistan	-	Asia
G99	Pakistan-16	16270	PGRI-Pakistan	Larkana	Sindh	Pakistan	-	Asia
G100	Pakistan-17	16355	PGRI-Pakistan	Dadu	Sindh	Pakistan	-	Asia
G101	Pakistan-18	16356	PGRI-Pakistan	Dadu	Sindh	Pakistan	-	Asia
G102	Pakistan-19	16357	PGRI-Pakistan	Karachi	Sindh	Pakistan	-	Asia
G103	Pakistan-20	16358	PGRI-Pakistan	Karachi	Sindh	Pakistan	-	Asia
G104	Pakistan-21	16359	PGRI-Pakistan	Gilgit	GB	Pakistan	-	Asia
G105	Pakistan-22	19233	PGRI-Pakistan	Gilgit	GB	Pakistan	-	Asia
G106	Pakistan-23	20920	PGRI-Pakistan	Islamabad	Federal Areas	Pakistan	-	Asia
G107	Pakistan-24	21933	PGRI-Pakistan	Karachi	Sindh	Pakistan	-	Asia
G108	Pakistan-25	24779	PGRI-Pakistan	Quetta	Balochistan	Pakistan	-	Asia
G109	Pakistan-26	27549	PGRI-Pakistan	Hyderabad	Sindh	Pakistan	-	Asia
G110	Pakistan-27	30698	PGRI-Pakistan	Hyderabad	Shindh	Pakistan	-	Asia
G111	Pakistan-28	35803	PGRI-Pakistan	Gakooch	Gilgit/Balistan	Pakistan	-	Asia
G112	Afganistan-4	7-T	CRIFC-Turkey	-	-	Afganistan	-	Asia
G113	Afganistan-5	9-T	CRIFC-Turkey	-	-	Afganistan	-	Asia
G114	China-8	27-T	CRIFC-Turkey	-	-	China	-	Asia
G115	China-9	29-T	CRIFC-Turkey	-	-	China	-	Asia
G116	Turkey-11	36-T	CRIFC-Turkey	-	Tarme	Turkey	-	Asia
G117	Turkey-12	37-T	CRIFC-Turkey	-	Tarme	Turkey	-	Asia
G118	Turkey-13	57-T	CRIFC-Turkey	-	Elbistan	Turkey	-	Asia
G119	Turkey-14	58-T	CRIFC-Turkey	-	Elbistan	Turkey	-	Asia
G120	Canada-1	74-T	CRIFC-Turkey	-		Canada	-	America
G121	Canada-2	75-T	CRIFC-Turkey	-		Canada	-	America
G122	USA-1	80-T	CRIFC-Turkey	-	Montana	USA	-	America
G123	Iran-8	116-T	CRIFC-Turkey	-		Iran	-	Asia
G124	USA-2	130-T	CRIFC-Turkey	-		USA	-	America
G125	USA-3	132-T	CRIFC-Turkey	-		USA	-	America
G126	Turkey-15	134-T	CRIFC-Turkey	-	Tarme	Turkey	-	Asia
G127	USA-4	149-T	CRIFC-Turkey	-	İdoha	USA	-	America
G128	Iran-9	152-T	CRIFC-Turkey	-		Iran	-	Asia
G129	USA-5	153-T	CRIFC-Turkey	-	İdoha	USA	-	America
G130	Iran-10	177-T	CRIFC-Turkey	-		Iran	-	Asia
G131	Turkey-16	277-T	CRIFC-Turkey	-	Tarme	Turkey	-	Asia

USDA: United States Department of Agriculture; PGRI: Plant Genetic Resources Institute; CRIFC: Central Research Institute for Field Crop;—Not known.

### 2.2. iPBS-retrotransposon PCR amplifications

Seventy iPBS-retrotransposon primers were initially screened using eight randomly selected accessions of safflower for PCR amplifications [25]. Out of the 70 iPBS-retrotransposon primers, 13 were found polymorphic and selected for PCR amplification, and produced strong bands (Table 2). A total reaction volume of 20 μL for PCR amplifications were comprised of 3 ng/ul template DNA, 2 μL dNTPs (Thermo Scientific), 0.2 μL U Taq DNA polymerase (Thermo Scientific), 3.2 μL primer, 2 μL 1x PCR buffer (Thermo Scientific), 2 μL MgCl_2_ and 7.6 μL distilled water. Reactions were performed in the sequence of denaturation at 95 ^o^C for 3 min, subsequently followed by 30 denaturation cycles at 95 ^o^C for 15 sec, annealing temperature 50–65 ^o^C for one minute depending upon the primer, and a final extension for five minute at 72 ^o^C [25]. The amplified fragments were electrophoresed on agarose gel 1.2% (w/v) using 0.5x TBE buffer at a constant voltage of 120 V for 230 minute. Staining of the gel was performed with ethidium bromide and visualized using UV Imager Gel Doc XR+ system (Bio-Rad, USA) light and photographed. A 100 bp+ DNA ladder was used as molecular weight marker.

**Table 2:** List of 13 iPBS-retrotransposon primers with their sequence and annealing temperature used to determine genetic diversity among 131 safflower accessions.

Primer name	Sequence	Annealing temperature (^o^C)
iPBS2252	TCATGGCTCATGATACCA	52
iPBS2376	TAGATGGCACCA	52
iPBS2377	ACGAAGGGACCA	53
iPBS2391	ATCTGTCAGCCA	52
iPBS2398	GAACCCTTGCCGATACCA	51
iPBS2228	CATTGGCTCTTGATACCA	53
iPBS2374	CCCAGCAAACCA	53
iPBS2399	AAACTGGCAACGGCGCCA	52
iPBS2401	AGTTAAGCTTTGATACCA	53
iPBS2239	ACCTAGGCTCGGATGCCA	52
iPBS2375	TCGCATCAACCA	52
iPBS2383	GCATGGCCTCCA	53
iPBS2392	TAGATGGTGCCA	52

### 2.3. Data analysis

**Fig 1:**
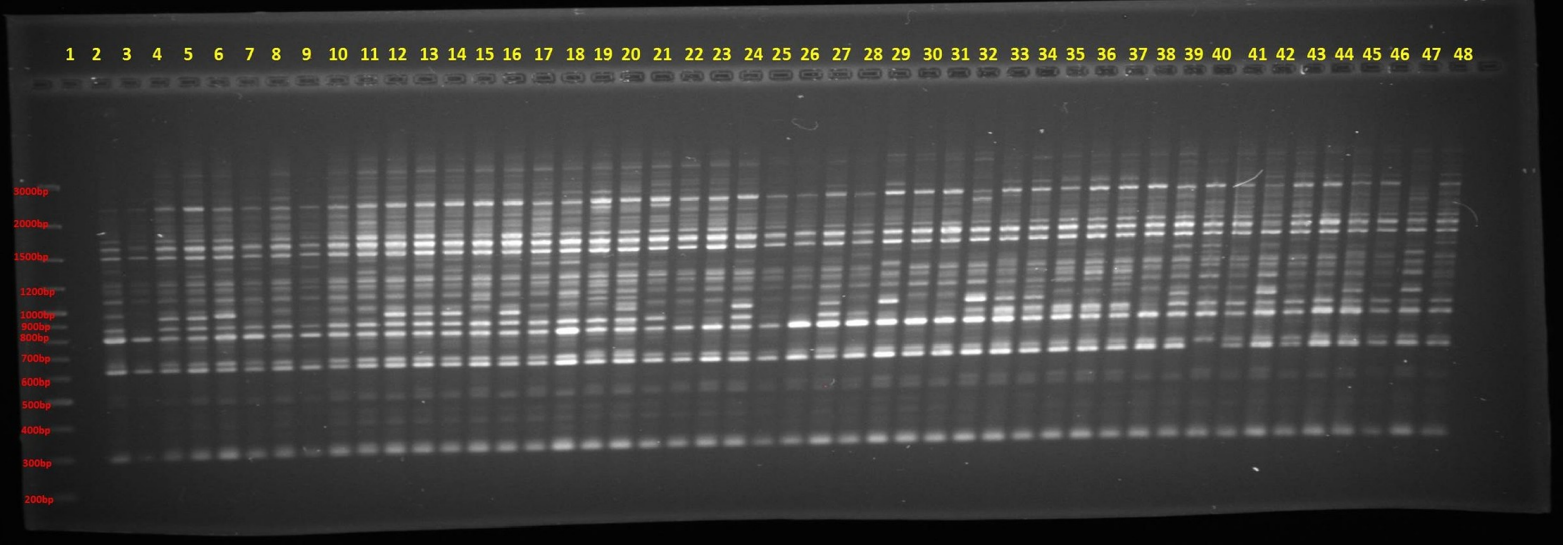
A representative gel imaging picture revealing genetic diversity among 131 safflower accessions using 13 iPBS-retrotransposon markers.

Strong, clear, and unambiguous bands were selected for scoring. iPBS-retrotransposon markers are dominantly inherited markers and were therefore scored using the binary system: 0 or 1, respectively, for the absence and presence of specific bands with respect to 100 bp+ DNA ladder (Fig 1). For individual iPBS-retrotransposon markers, PopGene ver. 1.32 [36] was used to estimate various important genetic diversity parameters including effective alleles number (Ne), Shannon's Information Index (I), and gene diversity (He) (Table 3). Polymorphism information content (PIC) was computed for each iPBS-retrotransposon marker following Baloch et al. [28] criteria. At the safflower samples level, the diversity metrics evaluated included the overall gene diversity (Ht), inbreeding coefficient (Fis) and the pair-wise FST (measure of genetic structure), all of which were determined using hierfstat R package [37] following the algorithms of Goudet et al. [38] and Yang, [39]. R statistical software was used to compute pairwise genetic distance (GDj) as measured by Jaccard’s coefficient [40]. The population structure was assessed using the Bayesian clustering model-based STRUCTURE software, unweighted pair group method with arithmetic mean (UPGMA), and Principle coordinate analysis (PCoA). The most suitable number of clusters (K subpopulations) was determined following the protocol of Evanno et al. [41] using STRUCTURE software. A total of ten independent runs were set for each K value, and for each run, the initial burn-in period was set to 500 with 500,000 MCMC (Markov chain Monte Carlo) iterations with no prior information on the origin of individuals. We plotted the clusters number (K) against logarithm probability relative to standard deviation (ΔK). Final assignment of individual accessions was based on the magnitude of the membership coefficient being greater than or equal to 50% as suggested by Habyarimana, [42] and Nadeem et al. [9]. R statistical software was used to analysis of molecular variance (AMOVA) for considering two main population strata: the model based structure and the country of origin of the accessions.

**Table 3:** List of various diversity parameters computed to evaluate genetic diversity among 131 safflower accessions using 13 iPBS- retrotransposon primers.

Primers	Total Bands	Polymorphic Bands	Polymorphism (%)	PIC	Ne	I	He	Ht
iPBS2252	20	15	75	0.432	1.2399	0.2666	0.1609	0.16092
iPBS2376	32	29	90.6	0.531	1.4461	0.4171	0.2746	0.26511
iPBS2377	36	29	80.6	0.781	1.4935	0.4578	0.3011	0.28914
iPBS2391	10	8	80	0.663	1.4672	0.4143	0.2745	0.27452
iPBS2398	22	20	90.9	0.316	1.3023	0.2901	0.1835	0.18353
iPBS2228	16	14	87.5	0.323	1.1813	0.1999	0.12	0.12005
iPBS2374	27	26	96.3	0.374	1.2904	0.313	0.1939	0.19393
iPBS2399	28	26	92.9	0.271	1.2293	0.2248	0.14	0.12747
iPBS2401	22	19	86.4	0.231	1.1578	0.1998	0.1117	0.07055
iPBS2239	28	26	92.9	0.623	1.324	0.3353	0.2084	0.18431
iPBS2375	22	20	90.9	0.587	1.4451	0.4055	0.2655	0.25677
iPBS2383	15	11	73.3	0.488	1.2603	0.2787	0.1693	0.12818
iPBS2392	17	14	82.4	0.582	1.5053	0.4372	0.2909	0.27281
Mean	22.69	19.77	86.1	0.477	1.334	0.3261	0.2073	0.19441
Total	295	275						

PIC: Polymorphism information content, Ne: effective alleles number, I: Shannon's Information Index, He: gene diversity, Ht: overall gene diversity

## 3. Results

### 3.1.iPBS-retrotransposon marker analysis and genetic diversity

Thirteen most polymorphic iPBS-retrotransposon primers produced a total of 295 clear and strong scorable bands with an average of 22.69 bands per primer across 131 safflower accessions. Out of the 295 scorable bands, 275 (93.22%) were polymorphic with an average of 19.77 bands per primer (Table 3). The highest (36) and lowest (10) number of scorable bands were observed for primers iPBS2377 and iPBS2391, respectively. The primers iPBS2376 and iPBS2377 revealed highest number of polymorphic bands (29) each and exhibited highest information content (PIC), while primer iPBS2391 revealed least number of polymorphic bands (8) and was least informative. The PIC value ranged from 0.23 (iPBS2401 primer) to 0.78 (iPBS2377 primer) with a mean of 0.48. Highest (1.51) and lowest (1.16) number of effective alleles were observed for primers iPBS2392 and iPBS2401, respectively with an average of 1.33 effective numbers of alleles. Similarly, maximum (0.46) and minimum (0.20) Shannon's information index was reported for primers iPBS2377 and iPBS2401 and iPBS2228 respectively, having an average value of 0.33. Highest (0.30) level of gene diversity was recorded for primer iPBS2377 while, lowest (0.11) level of gene diversity was observed for primer iPBS2401 with an average of 0.21. At the safflower accession samples level, the overall gene diversity (Ht), Fstatistic (Fst) and inbreeding coefficient (Fis) were 0.19, 0.21, and 1, respectively. The mean genetic diversity indices; observed number of alleles (1.86), effective number of alleles (1.34), Nei's gene diversity (0.21), Shannon's information index (0.33), and overall gene diversity (0.20) across four populations and one unclassified population were also determined (Table 4). Population A revealed observed number of alleles (1.68), effective number of alleles (1.28), Nei's gene diversity (0.17), Shannon's information index (0.28), and overall gene diversity (0.15). Population B revealed observed number of alleles (1.70), effective number of alleles (1.33), Nei's gene diversity (0.20), Shannon's information index (0.31), and overall gene diversity (0.19). Population C revealed observed number of alleles (1.63), effective number of alleles (1.26), Nei's gene diversity (0.16), Shannon's information index (0.26), and overall gene diversity (0.15). Population D revealed observed number of alleles (1.65), effective number of alleles (1.29), Nei's gene diversity (0.18), Shannon's information index (0.28), and overall gene diversity (0.17). Unclassified population revealed observed number of alleles (1.51), effective number of alleles (1.22), Nei's gene diversity (0.14), Shannon's information index (0.22), and overall gene diversity (0.13).

**Table 4:** Various diversity parameters computed to evaluate genetic diversity among 131 safflower across populations using 13 iPBS-retrotransposon primers.

Populations	Na	Ne	H	I	Ht	Mean Jaccard Genetic distance (GD)	GD Range
Population A	1.6814	1.2831	0.1748	0.2754	0.1498	0.222	0.05–0.339
Population B	1.6983	1.3255	0.1992	0.3096	0.1944	0.242	0.057–0.33
Population C	1.6305	1.2572	0.1616	0.2553	0.1459	0.238	0.126–0.357
Population D	1.6542	1.2931	0.1816	0.2840	0.1685	0.309	0.148–0.455
UP	1.5085	1.2150	0.1373	0.2176	0.1311	0.277	0.134–0.372
Overall	1.8644	1.3399	0.2106	0.3312	0.1971	0.288	0.05–0.507

Na: observed number of alleles, Ne: effective alleles number, I: Shannon's Information Index, h: gene diversity, Ht: overall gene diversity, UP: unclassified population

To clearly understand the broader picture of genetic diversity, pairwise genetic distance among 131 safflower accessions was measured with the Jaccard coefficient. The mean Jaccard genetic distance across the evaluated accessions was 0.288. The highest genetic distance (0.51) was observed between Turkey3 and Afghanistan4 accessions. Similarly, lowest genetic distance (0.05) was present between Afghanistan4 and Afghanistan5 accessions. Genetic distance was also calculated across the populations and mean genetic distance for population A (0.22), population B (0.24), population C (0.24), population D (0.31), and unclassified population (0.28).

Analysis of molecular variance (AMOVA) was carried out considering two main population strata: the model based structure and the country of origin of the accessions (Table 5). AMOVA revealed that the country of origin was not significant, while the model statistically significant effects on the molecular genotypic variability resulted from model-based structure (P = 0.005), country within model-based populations (P = 0.02), and model-based populations within country (P = 0.047). Variations between countries were not significant (P = 0.07), whereas variations within countries (P = 0.037) and between populations (P = 0.046) were significant (Table 6). The variations within and between populations explained 43 and 5 percent, respectively, of the genetic structure (Table 7). The country within population and the population within country explained 35 and 52 percent of the observed structure.

**Table 5:** Analysis of molecular variance (AMOVA) revealing genetic diversity in; (a) country within STRUCTURE populations, (b) populations within country.

			A			
Source	Df	SS	MS	F.Model	R2	Pr(>F)
Country	27	9417	348.78	1.4789	0.22364	0.152
country: group	26	14531	558.89	2.3698	0.34509	0.02*
Residuals	77	18160	235.84	0.43126		
Total	130	42108	1			
			B			
Source	Df	SS	MS	F.Model	R2	Pr(>F)
Structure	4	2177	544.35	2.3081	0.05171	0.005 **
group: country	49	21771	444.3	1.8839	0.51703	0.047 *
Residuals	77	18160	235.84		0.43126	
Total	130	42108			1	

“**” significance at the 0.1% nominal level and

“*” significance at the 1% nominal level; Country:group = country within STRUCTURE populations; Group:country = populations within country

**Table 6:** Analysis of molecular variance (AMOVA) revealing genetic diversity within the studied 131 safflower accessions.

Test	Obs	Std.Obs	Alter	Pvalue
Variations within samples	235.839	-1.9713	Less	0.037
Variations between samples	92.2606	1.69048	greater	0.07
Variations between group	-2.3109	2.06289	greater	0.046

**Table 7:** Analysis of molecular variance (AMOVA) revealing intra-genetic diversity within different Structure populations.

Source	Df	SS	MS	F.Model	R2	Pr(>F)
Populations	4	278.63	69.658	8.3981	0.21049	0.001 ***
Within populations	126	1045.11	8.295		0.78951	
Total	130	1323.74			1	

“***” corresponds to significance at the 0.05% nominal level

**Fig 2:**
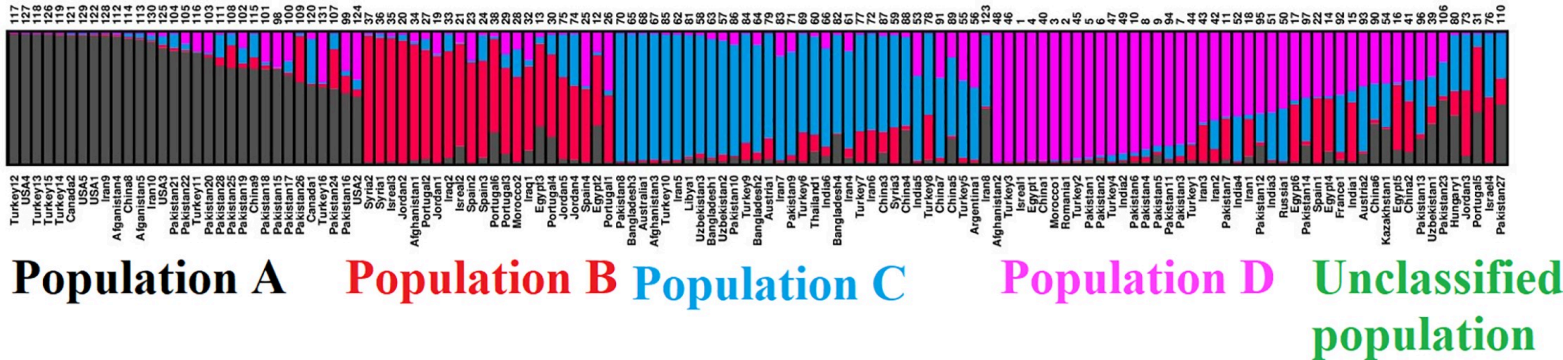
Structure-based clustering among 131 safflower accessions using 13 iPBS-retrotransposon markers.

**Fig 3:**
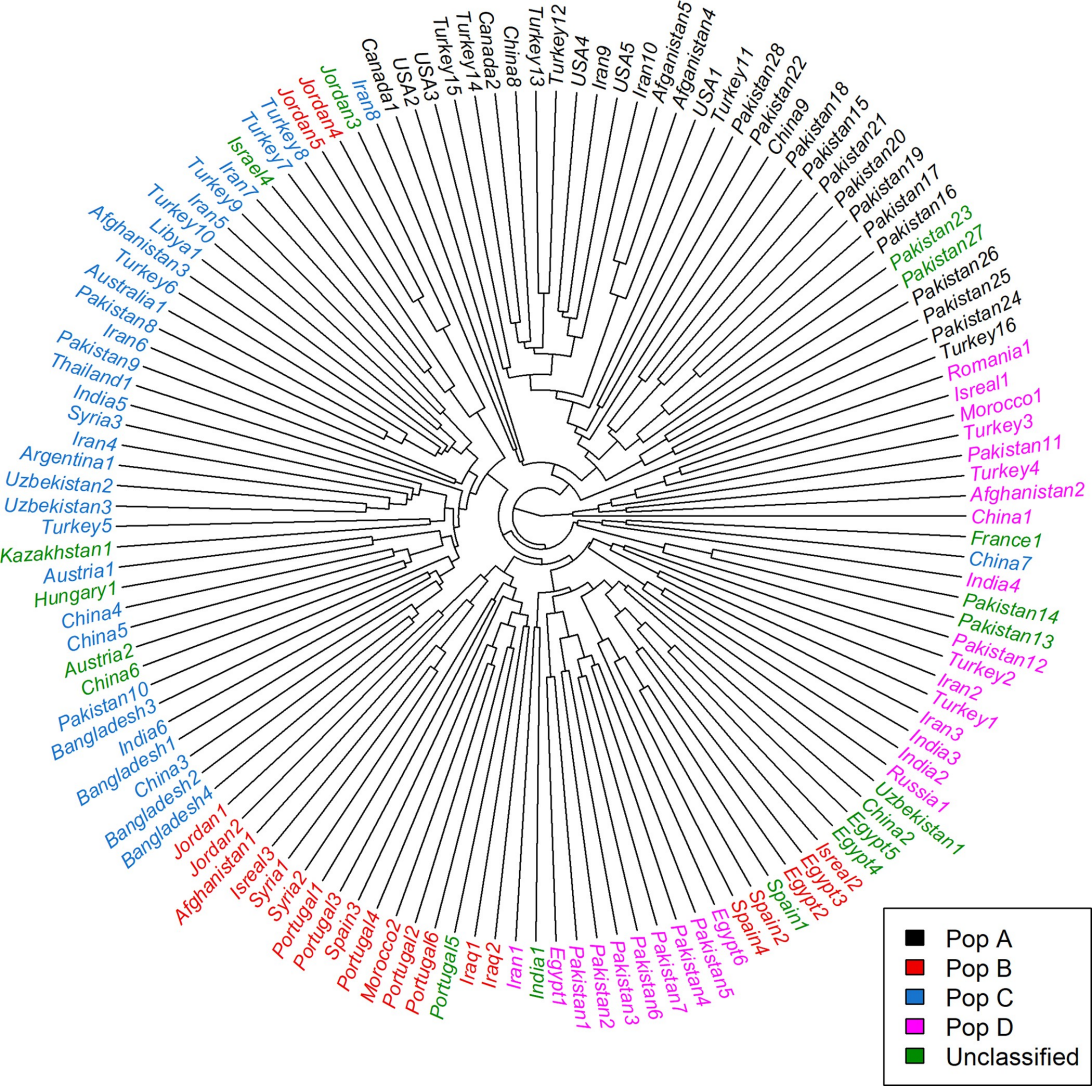
UPGMA based clustering among 131 safflower accessions using 13 iPBS-retrotransposon markers.

**Fig 4:**
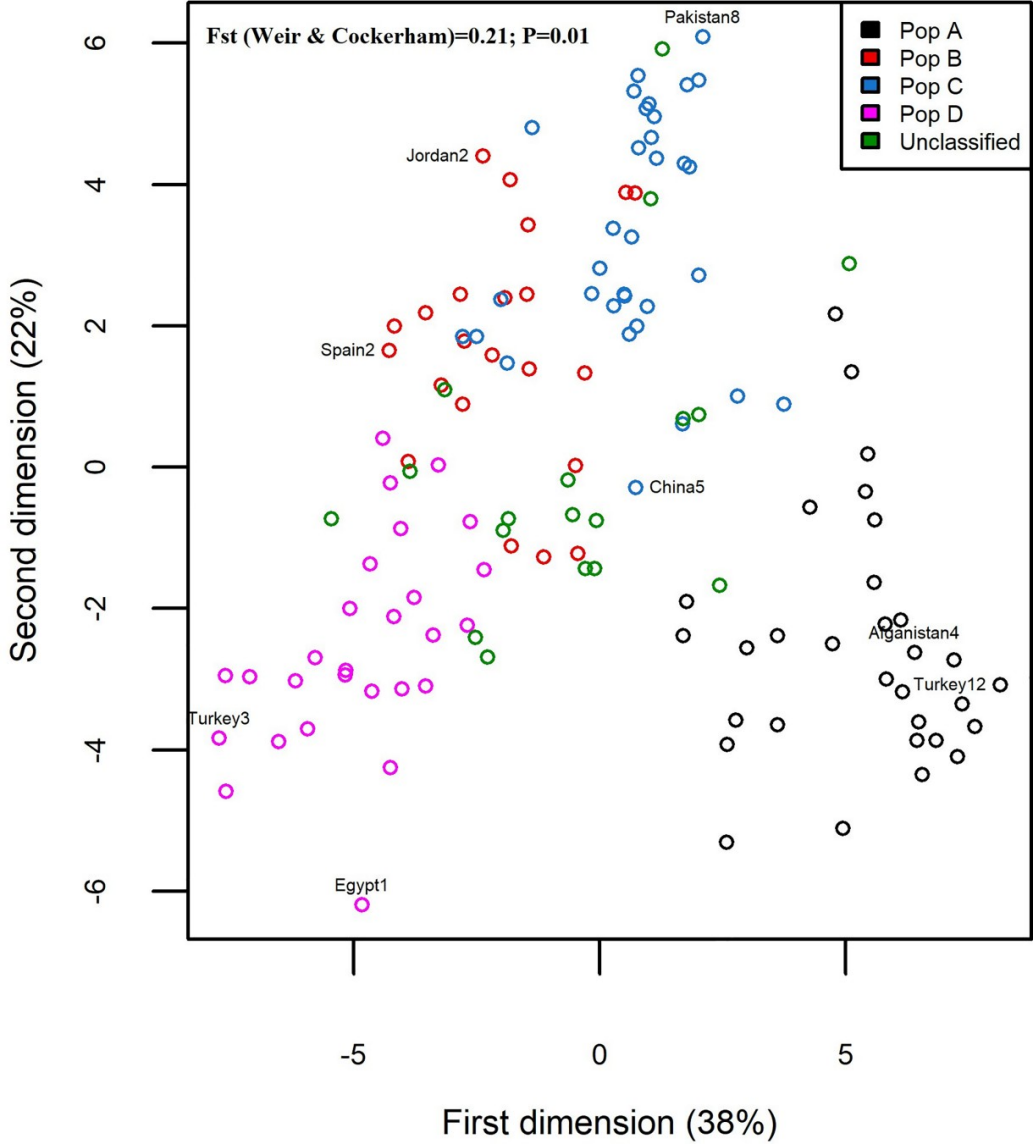
Principal coordinate analysis (PCoA) among 131 safflower accessions using 13 iPBS-retrotransposon markers.

In accordance with the observed most suitable goodness of fit (K = 4), the Bayesian clustering model implemented in STRUCTURE software divided the evaluated safflower accessions into four main populations; 31 accessions (23.66% of the total accessions) in the population A (black), 22 accessions (16.79% of the total accessions) in the population B (red), 33 accessions (25.19% of the total accessions) in the population C (blue), 27 accessions (20.61% of the total accessions) in the population D (pink) (Fig 2). Eighteen accessions (on the right-most end of the structure graph) did not reach the membership threshold (50%) and were named unclassified population. The UPGMA based clustering divided 131 safflower accessions into four main clusters corresponding to the four populations (populations A, B, C, D) identified using the model-based structure. The unclassified accessions were dispersed throughout the four populations, particularly in population D where 9 (50%) of the unclassified accessions clustered. With the UPGMA algorithm, two (Jordan4, Jordan5) and five (Israel2, Egypt2, Egypt3, Spain2, Spain4) population B accessions clustered with population D and population C, respectively. Similarly, relative to model-based clustering algorithm, UPGMA discrepantly clustered the accession Iran8 (population C) in population A (Fig 3). PCoA divide all accessions into four populations; A, B, C, and D similar to structure based clustering, with the unclassified accessions being dispersed particularly throughout populations B, C, and D (Fig 4).

## 4. Discussion

### 4.1. iPBS-retrotransposons in assessing genetic diversity of safflower panel

To the best of our knowledge, the present investigation represents the first attempt to elucidate the genetic diversity and population structure of safflower accessions at DNA level using iPBS-retrotransposons. It was observed that retrotransposons are abundant and widely distributed throughout plant genome [43] and huge amount of error-prone retroviral replications lead to the accumulation of these genetic variations [44–45]. iPBS based markers have been used greatly for fingerprinting and genetic diversity investigation in plants [26–35–46–47]. A total of 13 polymorphic iPBS- retrotransposon markers were used in this study to carry out genetic diversity in a panel of 131 safflower accessions from 28 different countries, and 295 clear and strong bands were recorded. The average number of bands per primer was 22.69 while, 275 (93.22%) out of 295 bands were polymorphic. Mean polymorphism found in this study was higher than that of Yang et al. [20], as they reported 82.7% polymorphism using ISSRs in 48 safflower accessions. Furthermore, Sehgal et al. [19] obtained even lower polymorphism levels of 57.6, 68.0, and 71.2% using RAPD, SSR and AFLP markers, respectively. Polymorphism is one of the key requirements to determine good quality genetic markers; therefore, iPBS markers satisfy this requirement in safflower.

Polymorphism information content (PIC) is a widely used metric of the usefulness of molecular markers [48]. The PIC was found higher (0.48) in this work than in the findings by Ambreen et al. [12], Ambreen et al. [13], Barati and Arzani [49], Derakhshan et al. [50], Hamdan et al. [33], and Lee et al. [17], all of whom used SSR markers to evaluate the genetic diversity in safflower. In their works, Houmanat et al. [51] found lower PIC value of 0.23 relative to this study, using ISSRs markers in safflower. These results clearly suggest that more diverse iPBS-retrotransposon markers loci can be identified and effectively used as a tool for assessing genetic diversity and other investigations relying on genetic variants. Maximum number of effective alleles is desirable because it represent the presence of higher level of genetic variations. Number of effective alleles (1.16 to 1.51) found in this work was in the similar range (1.29 to 1.72) to that of Panahi and Neghab [52] using ISSR markers to assess the genetic diversity in Iranian safflower germplasm. Similarly, Sung et al. [53] obtained lower range of effective number of alleles (1.02 to 1.09) than us using RAPD markers. Possible reason behind the presence of higher number of effective alleles in this study might be the differences of experimental materials used during evaluation and also the different molecular marker system. Shannon's information index usually distinguishes the level of available genetic diversity in a population, combining abundance and evenness. Kumar et al. [54] reported lower range of Shannon's information index (0.24 to 0.44) than this study using AFLP markers, highlighting the safflower accessions evaluated in this work were more diverse with genetic variants being more evenly distributed throughout the population. This was confirmed also by the level of gene diversity which was found higher than that of Ambreen et al. [12] and Pearl and Burke [18].

To know the genetic diversity more clearly, diversity metrics like; overall gene diversity (0.24), Fst (0.21), and Fis (1) were also computed. The Fst (a measure of genetic differentiation) obtained in this work was comparable with the findings of Ambreen et al. [12] as they obtained Fst in the range 0.08 to 0.29. On the other hand, Mokhtari et al. [55] obtained mean Fis value of 0.01 which is lower than that (1.00) presented in this work. Safflower is a self-pollinated crop, higher Fis values are therefore expected. In this study, the estimated Fst value (0.21) was higher than the variation explained by the genetic population as evaluated by the analysis of molecular variance (AMOVA). The difference of magnitude between the two metrics was expected as Fst accounted only for genetic populations as a source of variation, while AMOVA accounted for genetic populations and the provenance of the accessions. To understand the variations level more clearly, various diversity indices were calculated at the population’s level and population B was found superior by representing higher values for these diversity indices. On the other hand, unclassified population reflected lesser level of diversity by accounting lower values for these diversity indices.

The evaluation of pairwise genetic distance showed a mean of 0.288, with the highest genetic distance between accessions Turkey3 and Afghanistan4, followed by Afghanistan2 and Pakistan24 with respective distance values of 0.51 and 0.49. Greater similarity was found between Afghanistan4 and Afghanistan5 accessions showing least genetic distance of 0.05. One understandable reason behind the presence of maximum genetic similarity might be due to their origin from the common parents. To explore the genetic diversity more clearly, genetic distances were also calculated at the population level and mean maximum genetic distance was reflected by the population D and minimum was resulted by population A. Within populations, Turkey16 and China9 reflected maximum genetic distance and minimum was present between Afghanistan4 and Afghanistan5 accessions belonging to population A. Within population B, maximum genetic distance was observed between accessions Iraq1 and Jordan4, while minimum genetic distance was shown between accessions Jordan4 and Jordan5. Argentina1 and Iran8 were the two most distinct accessions reflecting maximum genetic distance in the population C and Australia1 and Turkey6 were found two most genetically similar accession of population C representing minimum genetic distance. Within population D, Turkey3 and Iran9 were most diverse accessions and Kazakhstan1 and Pakistan14 were two genetically distinct accessions belonging to unclassified population. Germplasm containing desirable plant traits can be usefully integrated in breeding programs to develop superior cultivars [24], particularly through controlled hybridizations involving genetically distant parental lines. The above four most diverse accessions identified in this work can be recommended as a candidate parents for future safflower breeding programs.

The analysis of molecular variance (AMOVA) was used to determine the pattern of the partition of the total gene diversity among and within populations, and the countries of origin [56]. AMOVA showed that most of genetic structure was explained by variations from individuals within populations, the genetic populations within countries and the countries within genetic populations. These findings are in agreement with Wodajo et al. [57], as they reported more within-population (98.9%) importance on genetic structure than among populations (1.1%) using ISSR markers to evaluate 70 safflower accessions from Ethiopia. The discrepancy in terms of the magnitude of variance components explained by the differing sources of variation included in the AMOVA model. The authors included in their model only the population as a source of variation, while in this work two sources of variation were considered including the population and the country of origin.

The model-based structure application proved more robust and informative in previous investigations [58–59]. Structure was therefore used in this work as a benchmark for clustering algorithms. Using structure, the 131 safflower accessions were partitioned into four main populations (A, B, C, and D), and 18 individuals with poor membership coefficients across clusters were considered unclassified population (Fig 2). A total of 31, 22, 33, 27, and 18 safflower accessions were found in populations A, B, C, D, and unclassified population, respectively. Population A comprised of 31 safflower accessions from Turkey, USA, Canada, Iran, Afghanistan, China, and Pakistan. This population represents the accessions from the Asian and North American regions. Population B consisted of 22 safflower accessions from different countries including Syria, Israel, Jordan, Afghanistan, Portugal, Spain, Morocco, Iraq, and Egypt. Population B contained the accessions from the Mediterranean countries and all clustering of these accessions together represents their genetic similarity. The 33 safflower accessions found in population C were collected from Pakistan, Bangladesh, Australia, Afghanistan, Turkey, Iran, Libya, Uzbekistan, Thailand, India, China, Syria, and Argentina. Population C comprised of accessions from the Asian and Mediterranean countries and clustering of accessions from both regions proposed the distribution of safflower from Mediterranean region to Asia through Turkey. Population D comprised of 27 safflower accessions from Afghanistan, Turkey, Israel, Egypt, China, Morocco, Romania, Pakistan, India, Iran, and Russia. The unclassified population composed of 18 safflower accessions from Pakistan, Spain, Egypt, France, India, Austria, China, Kazakhstan, Uzbekistan, Hungry, Jordan, Portugal, and Israel. Clustering of accessions from Mediterranean countries confirmed this region as center of origin for safflower especially Syria [3] and from this region, it is distributed to other parts of the world. Turkey, represents a great level of biodiversity, differentiation center among the continents, and played a vital role to connect the continents with each other [24].

On continents basis, population A clustered a total of 7 and 24 accessions belonging to American and Asian continents respectively. In population B, 3, 11 and 8 accessions originated from Africa, Asia and Europe, respectively. Population C comprised accessions from America (2), Asia (29), Europe (1), and Oceania (1). In population D most of the accessions originated from Asia (23), while a few accessions came from Africa (3) and Europe (1). The unclassified population contained genotypes mostly from Asia (11), while the other few came from Africa (2) and Europe (5) accessions also made divergence from above four populations by making their separate group. Clearly, the clustering based on molecular markers did not discriminate the origins of the safflower accessions evaluated in this work, which was also confirmed by the AMOVA inferences. Accessions from different countries clustered together, implying that kinship was more determinant for the population structure than the geographical provenance. In addition to sharing common parentage, similarities of accessions in same group during clustering might also be due to convergent evolution and selection [60]. It can therefore be inferred that populations from different geographical regions shared a great proportion of genetic diversity. The design of the experiment in this work cannot provide explanation of the observed predominance of Asian safflower accessions. However, the above countries of origin are part of the seven "centers of similarity" (the Far East, India-Pakistan, the Middle East, Egypt, Sudan, Ethiopia and Europe) as recognized by Knowles [5]. Safflower accessions from Afghanistan, Pakistan, Turkey, India, and particularly from China were found more diverse as they were present in all populations. The higher diversity observed in the Asian safflower accessions is a strong evidence of their wider adaptability, which is supported by the findings of Yang et al. [20] and Zhang [61].

In 1969, Knowles recognized the existence of seven safflower similarity centers across the world. Overall, the centers of similarity were represented by several accessions in this study. However, the molecular marker data used in this study did not provide much support to the above Knowles’s hypothesis on the similarity centers. Indeed accessions belonging to different similarity centers were clustered together. This lack of importance of similarity centers in defining molecular-based populations was reported in scientific literature [62]. In population A, the safflower accessions locally collected from Pakistan were mostly (12 accessions) part of the India-Pakistan similarity center. Also, six accessions from Turkey, two from Afghanistan, and two from Iran were present in this population and can be assigned to the Middle East similarity center. Population B comprised of safflower accessions from Syria (2), Israel (2), Jordan (4), Afghanistan (1), and Iraq (2) belonging to the Middle East similarity center. Similarly, population B contains safflower accessions from Spain (3), Portugal (5), and Morocco (1) which are part of the Europe similarity center. Population C exhibited safflower accessions from Afghanistan (1), Turkey (6), Iran (5), and Syria (1) revealing the Middle East similarity center. Also, population C contains accessions from Pakistan (3), Bangladesh (4), and India (2) showing the India-Pakistan similarity center. Population D revealed the India-Pakistan similarity center by containing accessions from India (3) and Pakistan (9). Population D also exhibits the Middle East similarity center because it contains accessions from Afghanistan (1), Turkey (4), Israel (1), and Iran (3). The unclassified population revealed the presence of Europe similarity center as it contains one accession from each country; Spain, France, Austria, Hungry, and Portugal. In the same way, India-Pakistan similarity center was also available in the unclassified population due to the presence of safflower accessions from Pakistan (4) and India (1). There is a still need for more research in order to shed more light on the safflower similarity centers at molecular level by collecting and evaluating accessions from all known similarity centers.

The investigation of genetic relationships between the 131 accessions using UPGMA clustering algorithm resulted in a clustering pattern comparable with the model-based algorithm with a few exceptions as two and five population B accessions clustered with population D and population C, respectively, and UPGMA discrepantly clustered the accession Iran8 (population C) in population A (Fig 3). Since these accessions displayed mostly full membership coefficients in model-based Structure, the discrepancy observed in UPGMA clustering approaches can be explained by its reduced resolution power relative to the model-based Structure [58–59].

Principal coordinate analysis (PCoA) greatly supported the structure based clustering of 131 safflower accessions using 13 iPBS-retrotransposon primers (Fig 4). The four populations were clearly distinguishable, and the unclassified population was disseminated throughout the other populations, particularly throughout populations B, C, and D. These light discrepancies between PCoA and model-based structure can derive from differing clustering resolution, with model-based structure exhibiting more resolution. Indeed, 40% of the variation in the overall genetic structure was not accounted for by the first two PCoA dimensions presented in this work. The above-mentioned misclassifications of accessions in the principal coordinate space can be explained by the existence of genomic admixture. PCoA analysis revealed the same pattern of distribution of similarity centers as identified by structure based analysis. Population A, B, and D exhibited the Middle East similarity centers as they contain safflower accessions from Turkey, Afghanistan, Iran, Syria, Israel, Jordan, and Iraq. Population C comprised of India-Pakistan similarity center by containing safflower accession from India, Pakistan, and Bangladesh. Europe similarity center is present in population B and in the unclassified population of PCoA based analysis. It suggests more research work regarding the confirmation of safflower similarity centers at molecular level. Overall, iPBS-retrotransposons revealed a good spectrum of genome diversity in safflower and the explored genetic diversity can be used in future safflower breeding programs. As iPBS-retrotransposon marker system demonstrated competitive results in this work and in previous investigations, it is warranted to focus further attention on collecting and evaluating safflower germplasm at molecular level using iPBS-retrotransposons as an important tool for enhancing productivity. To contribute to the yet unending discussion on the safflower similarity centers, a robust sampling techniques including random sampling without replacement can be implemented on the accessions in major world safflower seed repositories; the sampled materials can be evaluated using clustering algorithms such as those implemented in this work.

## 5. Conclusion

A good level of genetic diversity was identified among 131 safflower accessions. The importance of genetic populations on the genetic structure was significant, but its magnitude was lesser than the importance the variations of individuals within genetic populations. The provenance of the samples showed no effects on the genetic structures in the 131 accessions. Our results most probably obey the seven similarity centers hypothesis of safflower but still there is need to conduct further research works to confirm these similarity centers at the molecular level. Generally, safflower accessions from Asian countries like Afghanistan, Pakistan, China, Turkey, and India were found diverse. Specifically, among 131 safflower germplasm, accessions Turkey3, Afghanistan4, Afghanistan2, and Pakistan24 were found most diverse at molecular level and might be recommended as a candidate parents for future safflower breeding programs.
